# Letter to the editor regarding the article “The effects of virtual reality technology on reducing pain in wound care: A meta‐analysis and systematic review”

**DOI:** 10.1111/iwj.13905

**Published:** 2022-07-31

**Authors:** Rong Hu, Zhongmin Fu, Ning Ning, Fang He, Yeping Li

**Affiliations:** ^1^ Mianyang Central Hospital School of Medicine, University of Electronic Science and Technology of China Mianyang PR China; ^2^ West China School of Nursing Sichuan University Chengdu PR China; ^3^ Department of Orthopedics, West China Hospital Sichuan University Chengdu PR China

Dear Editor,

We read with great interest the review by He et al^1^ about the effects of virtual reality technology on reducing pain in wound care. We highly appreciate their contribution to this important topic. Coincidentally, we did a systematic review and meta‐analysis on a similar topic in January 2022. After careful review, there are several concerns worth communicating.

First, this systematic review claims its focus on adult patients who have accepted wound care in the methods section. However, three of the referred studies^2^, ^3^, ^4^ reported other cases (children,^2^ patients aged 9 to 40 years,^3^ teenagers aged 11to18 years^4^) excluding adult cases. Tumi et al^5^ found statistically significant differences in pain sensitivity response between old (62.2 ± 3.4 to 79 ± 4 years) and younger adults (22 ± 1.5 to 39.1 ± 8.8 years). Younger children (6 to 8.12 years) were more sensitive to noxious stimuli than older children. The authors do not state that these non‐adult cases were excluded from their study. These three studies are not suitable for this meta‐analysis, which impairs the certainty of the conclusions.

Second, in the “Statistical methods” part of the “Methods” section, there was one writing mistake: “If *p* > 0.1, I^2^ < 50% indicated that there was no heterogeneity among the studies, and a fixed effect model was selected for analysis; if *p* ≤ 0.1, I^2^ ≥ 50% indicated that there was no heterogeneity among the studies”, the “no heterogeneity” appears twice.

Third, the figure “Risk of bias summary” suggests that the quality of RCTs exacerbates our concerns. We suggest the Grading of Recommendations Assessment, Development, and Evaluation (GRADE) approach to assess the certainty of evidence. Another concern is about the high heterogeneities in the results (I^2^ > 80% was found in 6 outcomes). We recommend further subgroup analysis according to surgery type to explain the source of heterogeneity.

Finally, trial sequential analysis (TSA) can minimise the type 1 errors and type 2 errors caused by small sample sizes. It would have been better if the authors had presented the results with TSA and included data to determine whether the evidence was reliable and conclusive. In our study, trial sequential analysis was performed for the Visual Analog Scale (VAS), the required information size (RIS) for the primary outcome was based on a type I error of 5% and a power of 80%. Only the cumulative Z‐curve crossed trial sequential monitoring boundaries (TSMB), and the anticipated intervention effect had firm evidence. Otherwise, evidence was rated as absent. In our study, the result of VAS suggests that the Z curve failed to cross the TSMB (TSA adjusted CI: −2.36 to −0.35; Figure 1) and the RIS (*n* = 3156) was not reached. Therefore, more high‐quality studies are needed to confirm VR therapy as an auxiliary analgesic intervention on the pain level in wound care.

**FIGURE 1:**
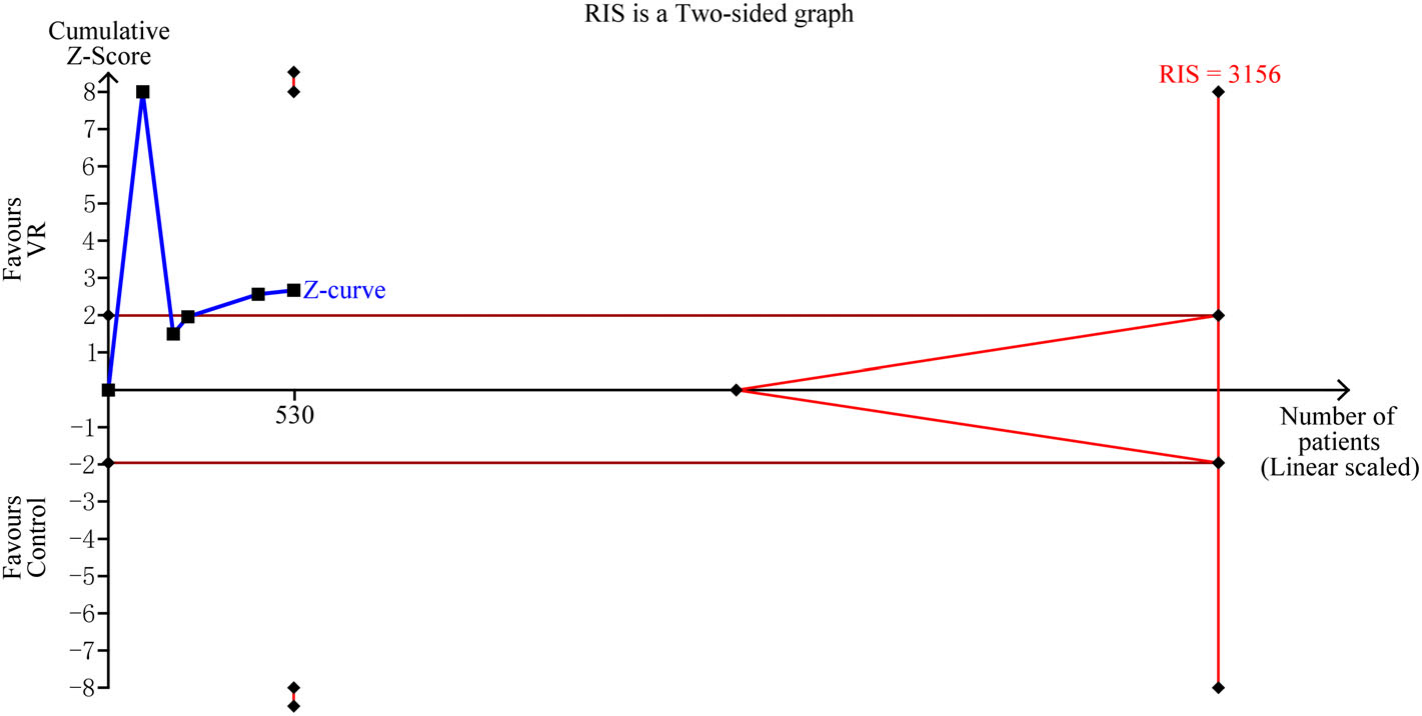
Trial sequential analysis of mean VAS score in wound care: blue line indicates the trend of the pooled estimates with the addition of data from each clinical trial.

We respectfully appreciate that the authors provided us with an important meta‐analysis.

## CONFLICT OF INTEREST

The authors declare that there is no conflict of interest.

## Data Availability

The data that support the findings of this study are available from the corresponding author upon reasonable request.
